# Safety and efficacy of infliximab in the treatment of refractory uveoretinitis in Behçet’s disease: a large-scale, long-term postmarketing surveillance in Japan

**DOI:** 10.1186/s13075-018-1793-7

**Published:** 2019-01-05

**Authors:** Shigeaki Ohno, Itsuro Umebayashi, Miyuki Matsukawa, Takashi Goto, Toshiro Yano

**Affiliations:** 1 Department of Ophthalmology, Faculty of Medicine and Graduate School of Medicine, Hokkaido University, N 15, W 7, Kita-ku, Sapporo, Hokkaido 060-8638 Japan; 2 Department of Ophthalmology, Aishin Memorial Hospital, 1-15, N27, E1, Higashi-ku, Sapporo, Hokkaido 065-0027 Japan; 3 Ikuyaku. Integrated Value Development Division, Mitsubishi Tanabe Pharma Corporation, 3-16-89, Kashima, Yodogawa-ku, Osaka, Japan; 4 Ikuyaku. Integrated Value Development Division, Mitsubishi Tanabe Pharma Corporation, 3-2-10, Dosho-machi, Chuo-ku, Osaka, Japan

**Keywords:** Behçet’s disease, Uveoretinitis, Biological therapies, Infliximab, Postmarketing surveillance, Safety, Efficacy, Associated factor

## Abstract

**Background:**

Infliximab, an anti-tumor necrosis factor-alpha antibody, has been reported to have excellent efficacy for refractory uveoretinitis in Behçet’s disease (RUBD), and was approved for this indication in Japan. However, the long-term safety profile and efficacy in real-world clinical settings in patients with RUBD have not been fully clarified. The BRIGHT study, a prospective, large-scale, long-term postmarketing surveillance (PMS) study, was conducted to investigate the long-term safety and efficacy of infliximab in Japanese patients with RUBD.

**Methods:**

All patients with RUBD who started infliximab treatment between January 2007 and January 2010 were enrolled. Safety was evaluated every 6 months for up to 24 months after initiation of therapy in 656 patients, and efficacy was evaluated in 650 patients. Patient characteristics were compared using the chi-square or Fisher’s exact test. The frequency of ocular attacks before and after infliximab treatment was compared using the Wilcoxon signed-rank test. Independent associated factors for safety or efficacy were identified using multiple logistic regression analysis. A two-sided *p* value <0.05 was considered significant.

**Results:**

Among the 656 patients evaluated for safety, 555 (84.6%) completed the 24-month study period. The incidence of adverse drug reactions (ADRs) and serious ADRs were 32.32% and 6.10%, respectively, and the safety profile was comparable to that of Japanese PMS of infliximab for other diseases. The most common ADRs and serious ADRs were infections (11.89% and 3.66%). Tuberculosis was reported in two patients, and *Pneumocystis jirovecii* in one. Identified independent associated factors for infections were comorbid respiratory disease, history of allergic disease, and concomitant use of glucocorticoids. Although infusion reactions were observed in 11.13% of patients, most were non-serious. The response rate at 24 months by physician global assessment was 80.7%. Median frequency of ocular attacks per 6 months significantly decreased compared with that before infliximab treatment (2.0 to 0.0), and corrected visual acuity was maintained during the study.

**Conclusions:**

Infliximab treatment had good tolerability and efficacy in Japanese patients with RUBD in this large-scale, long-term PMS. Infliximab treatment seemed to be a good treatment option for RUBD in real-world clinical settings.

**Trial registration:**

UMIN Clinical Trials Registry, UMIN000027733. Retrospectively registered on 6 June 2017.

**Electronic supplementary material:**

The online version of this article (10.1186/s13075-018-1793-7) contains supplementary material, which is available to authorized users.

## Background

Behçet’s disease (BD), common along the coast of the Mediterranean Sea and the Silk Road extending to Japan, is a systemic inflammatory condition of unknown etiology characterized by recurrent aphthous ulcers, skin lesions, genital ulcers and ocular lesions [1]. Ocular inflammation in BD causes uveoretinitis, which in severe cases results in irreversible damage to the retina and optic nerve, and blindness. Current treatments for BD uveoretinitis include glucocorticoids, cyclosporine and other immunosuppressants. In particular, cyclosporine exerts potent immunosuppressive activity via the suppression of T-cell function, and various guidelines recommend its use for the treatment of BD uveoretinitis [2]. However, given the adverse drug reactions (ADRs) associated with cyclosporine, including nephrotoxicity and central nervous system effects, and the existence of non-responders to cyclosporine, a novel therapeutic agent is desirable.

Tumor necrosis factor-alpha (TNF) has been implicated in the pathogenesis of BD. TNF mediates murine susceptibility to experimental autoimmune uveoretinitis (EAU) [3], while administration of anti-TNF antibodies effectively suppresses the induction of EAU [4]. Further, monocyte-derived TNF levels are elevated in patients with ocular symptoms of BD [5].

Infliximab (IFX), an anti-human TNF monoclonal antibody, neutralizes TNF activity and binds to transmembrane TNF-producing cells, resulting in their apoptosis [6, 7]. IFX has shown good efficacy against various inflammatory diseases, including inflammatory bowel disease, rheumatoid arthritis (RA), and psoriasis [8–15], and has been administered to close to 3 million patients with these diseases. In addition, it has also been reported to be useful in patients with refractory uveoretinitis of BD (RUBD) [15–17]. Based on findings of early phase II clinical trials (*n* = 13) [18], long-term trials (*n* = 9), and phase III trials (*n* = 12) in Japanese patients with RUBD (Mitsubishi Tanabe Pharma Corporation internal data), IFX was approved for this indication in Japan for the first time in the world in January 2007.

Here, to investigate the safety and efficacy of IFX treatment in RUBD in clinical practice, we conducted a prospective, large-scale, long-term postmarketing surveillance (PMS) study of all IFX-treated patients in Japan (the BRIGHT study: PMS in patients with Behcet’s disease at Remicade (Infliximab) treatment; lonG-term safety/efficacy for sigHT-threating uveoretinitis).

## Methods

### Study design and patients

This PMS study (UMIN000027733) included all patients with RUBD starting treatment with IFX at 215 Japanese medical institutions between January 2007 and January 2010. These patients had not responded to conventional therapy, including glucocorticoids, cyclosporine, colchicine (which is often used and recommended for treatment of RUBD in Japan) [19, 20]. and other immunosuppressants. The evaluation period was 24 months in total, and safety and efficacy data were prospectively collected at the end of 6, 12, and 24 months during the evaluation period.

### Procedures

Prior to initiating IFX, patients were examined for the presence of tuberculosis (TB) by inquiring about past medical and family history, and tuberculin skin tests (TST) and radiographic chest examinations. Anti-TB agents were administered in suspected cases of TB infection.

Patients received commercially available IFX (Remicade®) at 5 mg/kg body weight at weeks 0, 2, and 6, and then every 8 weeks thereafter (in accordance with the indication for RUBD in Japan) for the entire 24 months of the study. Information on ADRs were recorded, including type, date of diagnosis, severity, and outcome, and ADRs were classified according to the preferred terms and system organs class (SOC) of the Medical dictionary for regulatory activities (MedDRA/J, version 19.1, http://www.meddra.org/sites/default/files/guidance/file/intguide_19_1_japanese.pdf, Japanese article, last accessed December 25, 2018). Infusion reactions (IRs) were defined as any ADRs occurring during or within 2 h after the completion of any infusion.

The clinical response was evaluated based on physician global assessment (PGA) using a 4-point scale (improved, slightly improved, unchanged, and worsened). PGA was used for analysis of clinical response as most patients enrolled in this study were anticipated to have severe symptoms prior to this study, resulting in inaccurate evaluation by using ocular severity (severe/moderate/mild). PGA was evaluated by each physician by comparing the ocular symptoms before and at 3-month intervals during IFX treatment, with “improved”, or “slightly improved” defined as the patient having a PGA response.

The number of ocular attacks was evaluated every 6 months in patients in whom ocular attacks within 6 months prior to initiation of IFX were evaluated. To precisely evaluate efficacy in reducing ocular attacks and associated factors, we evaluated efficacy in patients who had no history of IFX treatment and had at least one episode of ocular attack within 6 months prior to IFX treatment. Corrected visual acuity was examined on a monthly basis. Final efficacy (at 24 months) was evaluated using both as-observed and last observation carried forward (LOCF) analysis.

In addition, changes in the dose of concomitant cyclosporine, glucocorticoids, and colchicine, and the percentage of patients receiving these agents were examined.

### Statistical analysis

Continuous data were summarized using descriptive statistics. Discrete data were summarized based on the number and percentage values for each category. Patient characteristics were compared using the chi-square test or Fisher’s exact test. The frequency of ocular attacks within 6 months before and after IFX treatment was compared using the Wilcoxon signed-rank test. Independent correlations among factors associated with the development of any infections, or response in PGA (improved or slightly improved), were identified using multiple logistic regression analysis. Multiple logistic regression analysis of the absence of ocular attacks after the initiation of IFX treatment included the number of ocular attacks in the 6 months prior to IFX treatment as an explanatory variable. All multiple logistic regression analyses were conducted using a stepwise selection process. A significance level of 5% and two-sided 95% confidence intervals (CIs) were defined. Statistical analyses were performed using SAS software version 9.1.3 (SAS Institute Japan Ltd., Tokyo, Japan).

## Results

### Patients

**Fig. 1:**
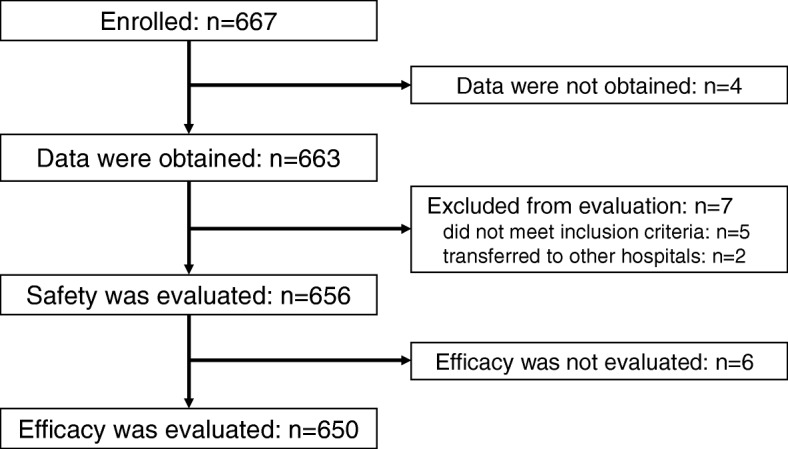
Study profile

In total, 667 patients in whom IFX treatment was initiated were enrolled in this study, and case report data were obtained for 663 patients. Of these 663 patients, 7 were excluded due to various reasons such as failure to meet the inclusion criteria (*n* = 5) or hospital transfer (*n* = 2), leaving 656 patients who could be evaluated for safety. Efficacy was evaluated in 650 patients for whom efficacy data were available (Fig. 1).

The demographics and disease characteristics of patients who participated in this study are shown in Table 1. With regard to 656 patients monitored for drug safety, 23.5% were female, mean age was 40.1 years, with respective mean disease durations of BD and uveoretinitis of 7.46 and 6.63 years, respectively.

**Table 1:** Patient characteristics

	Value in patients (*n* = 656)
Sex (female)	154 (23.5)
Age, years	
mean (SD), [range]	40.1 (12.1), [10–78]
< 15	3 (0.5)
≥ 15–< 25	46 (7.0)
≥ 25–< 35	189 (28.8)
≥ 35–< 45	219 (33.4)
≥ 45–< 55	109 (16.6)
≥ 55–< 65	65 (9.9)
≥ 65	25 (3.8)
Disease duration of BD, years	
mean (SD), [range]	7.46 (6.80), [0.1–49.0]
< 5	268 (40.9)
≥ 5–< 10	159 (24.2)
≥ 10–< 15	95 (14.5)
≥ 15	77 (11.7)
unknown	57 (8.7)
Disease duration of uveitis, years	
mean (SD), [range]	6.63 (5.91), [0.0–36.0]
< 5	294 (44.8)
≥ 5–< 10	166 (25.3)
≥ 10–< 15	86 (13.1)
≥ 15	60 (9.1)
unknown	50 (7.6)
Severity of ocular symptoms	
severe	408 (62.2)
moderate	201 (30.6)
mild	36 (5.5)
unknown	11 (1.7)
History of TB infection	35 (5.3)
Prophylactic anti-TB drug	242 (36.9)
History of HBV infection	6 (0.9)
unknown	1 (0.2)
History of allergic disease	38 (5.8)
History of IFX use	31 (4.7)
Extraocular symptoms of BD	
any symptoms	592 (90.2)
oral aphthous ulcers	534 (81.4)
genital ulcers	226 (34.5)
skin lesions	392 (59.8)
arthritis	171 (26.1)
epididymitis	30 (4.6)
central nervous system lesions	64 (9.8)
intestinal tract lesions	67 (10.2)
vascular lesions	20 (3.0)
other symptoms	26 (4.0)
unknown	5 (0.8)
Comorbidity	
any comorbidity	360 (54.9)
respiratory disease	10 (1.5)
hepatic disease	35 (5.3)
cardiac disease	10 (1.5)
ocular disease	185 (28.2)
kidney disease	23 (3.5)
malignancy	2 (0.3)
diabetes mellitus	31 (4.7)
other diseases	200 (30.5)
Drug use in prior 6 months	
cyclosporine	251 (38.3)
dose, median (Q1, Q3), mg/day, *n* = 242^a^	168.24 (120.00, 200.00)
glucocorticoids	239 (36.4)
dose, median (Q1, Q3), mg/day, *n* = 235^a^	13.07 (7.50, 20.00)
colchicine	328 (50.0)
dose, median (Q1, Q3), mg/day, *n* = 325^a^	1.00 (1.00, 1.00)
Infliximab dose, median (Q1, Q3) [range], mg/kg	5.00 (5.00, 5.00) [3.4–6.4]

Data are number (percentage), unless otherwise described

*BD* Behçet’s disease, *TB* tuberculosis, *HBV* hepatitis B virus, *Q1* 1st quartile, *Q3* 3rd quartile

^a^Number of patients in whom dosage data were obtained

Many patients had severe uveoretinitis (62.2%). The majority had extraocular involvement (90.2%), the most common of which was recurrent oral aphthous ulcers (81.4%), followed by skin lesions (59.8%) and genital ulcers (34.5%). Cyclosporine, glucocorticoids, and colchicine were used in 38.3%, 36.4%, and 50.0% of patients, respectively, within 6 months prior to IFX treatment. Median (1st quartile (Q1), 3rd quartile (Q3)) IFX dosage was 5.00 (5.00, 5.00) mg/kg body weight, and the rates of < 5.00 mg/kg, 5.00 mg/kg, and > 5.00 mg/kg were 5.9%, 85.2%, and 8.8%, respectively.

The number of patients followed for 6, 12, and 18 months were 624, 597, and 565, respectively, and 555 patients completed the study period. The main reasons for discontinuation were transfer/failure to attend hospital (*n* = 56), followed by the occurrence of adverse events (*n* = 28). Mean (SD) length of observation was 666.9 (172.1) days, and median (Q1, Q3) number of IFX administrations was 15.0 (14.0, 15.0) times.

### Safety

ADRs and serious ADRs were observed in 32.32% (212/656) and 6.10% (40/656) of patients, respectively, during the study period (day 0–730, Table 2). Occurrence of ADRs tended to be higher at the initiation of treatment, and no increase was observed thereafter. The incidence of all ADRs and serious ADRs classified using the MedDRA/J SOC are also described in Table 2. “Infections and infestations” was the most common ADR (11.89%), followed by “skin and subcutaneous tissue disorders” (9.60%). The most common serious ADRs were also Infections and infestations (3.66%), while the occurrences of other serious ADRs < 1.0%. Major infections were nasopharyngitis (*n* = 10), gastroenteritis (*n* = 7), herpes zoster, pharyngitis, and pneumonia (each *n* = 6); and major serious infections were pneumonia (*n* = 4), cellulitis, gastroenteritis, septic shock, and sinusitis (each *n* = 2).

**Table 2:** Safety profile of infliximab therapy classified by system organ class (SOC)

	Day 0–180 (*n* = 656)	Day 181–365 (*n* = 624)	Day 366–545 (*n* = 597)	Day 546–730 (*n* = 565)	All period (day 0–730) (*n* = 656)*
Any ADRs	122 (18.60)	73 (11.70)	44 (7.37)	40 (7.08)	212 (32.32)
Blood and lymphatic system disorders	0	0	1 (0.17)	0	1 (0.15)
Cardiac disorders	2 (0.30)	1 (0.16)	0	0	3 (0.46)
Eye disorders	3 (0.46)	2 (0.32)	1 (0.17)	0	6 (0.91)
Gastrointestinal disorders	8 (1.22)	2 (0.32)	1 (0.17)	1 (0.18)	12 (1.83)
General disorders and administration site conditions	15 (2.29)	7 (1.12)	4 (0.67)	5 (0.88)	31 (4.73)
Hepatobiliary disorders	4 (0.61)	1 (0.16)	0	1 (0.18)	5 (0.76)
Immune system disorders	2 (0.30)	0	1 (0.17)	0	3 (0.46)
Infections and infestations	35 (5.34)	25 (4.01)	14 (2.35)	13 (2.30)	78 (11.89)
Injury, poisoning and procedural complications	7 (1.07)	5 (0.80)	6 (1.01)	5 (0.88)	19 (2.90)
Investigations	18 (2.74)	5 (0.80)	3 (0.50)	5 (0.88)	30 (4.57)
Metabolism and nutrition disorders	1 (0.15)	0	1 (0.17)	0	2 (0.30)
Musculoskeletal and connective tissue disorders	3 (0.46)	3 (0.48)	2 (0.34)	1 (0.18)	8 (1.22)
Neoplasms benign, malignant and unspecified	1 (0.15)	0	1 (0.17)	1 (0.18)	3 (0.46)
Nervous system disorders	7 (1.07)	1 (0.16)	1 (0.17)	0	9 (1.37)
Respiratory, thoracic and mediastinal disorders	12 (1.83)	13 (2.08)	5 (0.84)	5 (0.88)	29 (4.42)
Skin and subcutaneous tissue disorders	39 (5.95)	20 (3.21)	16 (2.68)	7 (1.24)	63 (9.60)
Serious ADRs	19 (2.90)	8 (1.28)	6 (1.01)	8 (1.42)	40 (6.10)
Blood and lymphatic system disorders	0	0	0	0	0
Cardiac disorders	1 (0.15)	0	0	0	1 (0.15)
Eye disorders	1 (0.15)	1 (0.16)	0	0	2 (0.30)
Gastrointestinal disorders	1 (0.15)	0	0	0	1 (0.15)
General disorders and administration site conditions	0	0	1 (0.17)	1 (0.18)	2 (0.30)
Hepatobiliary disorders	0	0	0	0	0
Immune system disorders	0	0	1 (0.17)	0	1 (0.15)
Infections and infestations	11 (1.68)	7 (1.12)	2 (0.34)	5 (0.88)	24 (3.66)
Injury, poisoning, and procedural complications	1 (0.15)	0	0	0	1 (0.15)
Investigations	0	0	0	1 (0.18)	1 (0.15)
Metabolism and nutrition disorders	0	0	0	0	0
Musculoskeletal and connective tissue disorders	0	0	1 (0.17)	0	1 (0.15)
Neoplasms benign, malignant and unspecified	1 (0.15)	0	0	1 (0.18)	2 (0.30)
Nervous system disorders	1 (0.15)	0	1 (0.17)	0	2 (0.30)
Respiratory, thoracic and mediastinal disorders	3 (0.46)	0	1 (0.17)	0	4 (0.61)
Skin and subcutaneous tissue disorders	1 (0.15)	0	0	0	1 (0.15)

Data are number (percentage)

ADRs, adverse drug reactions

^a^All patients’ data were used in the evaluation safety including those who did not complete this study

Tuberculosis (TB) was observed in two patients (0.30%) who had not received anti-TB therapy despite a positive tuberculin skin test (TST) (diameter ≥ 10 mm); one had pulmonary TB and intrathoracic lymph node involvement and the second had disseminated TB. Both cases resolved after anti-TB treatment. No TB case was observed in patients who received prophylactic anti-TB drugs. Opportunistic infections were observed in three patients, including cytomegalovirus infection, septic shock, and pneumocystis pneumonia (each *n* = 1). All cases resolved after anti-viral, anti-microbial, or anti-fungal treatment.

Infusion reactions (IRs) and serious IRs were observed in 73 (11.13%) and 3 (0.46%) of patients, respectively (Additional file 1: Figure S1). IRs occurred from the 1st to the 16th infusion at a rate of 0.37–2.15% for each infusion, with no tendency towards more IRs at an earlier stage of treatment. Serious IRs (three patients, five incidences) were observed on the 4th, 5th, 8th, 9th, and 14th infusions. Among 71 patients who developed non-serious IRs, 66 (93.0%) received re-infusion of IFX, among whom 1 subsequently developed a serious IR, 21 developed non-serious IRs, and 44 did not develop IRs. Both patients who developed serious IRs received re-infusion; one again developed a serious IR, while the second did not develop IRs thereafter (Additional file 1: Figure S1).

Lupus-like syndrome was observed in one patient who had an increase in anti-nuclear antibody but no symptoms. Demyelinating disorder was observed in one patient, and malignancies were observed in two (lung neoplasm and papillary thyroid cancer); their symptoms were resolved after the appropriate treatment. No fatal ADRs was observed during the study.

### Efficacy

Efficacy was evaluated in 650 patients (Fig. 1). Although PGA data were obtained for 642 patients, data were “undeterminable” in 19 patients. Therefore, PGA data were evaluated for the remaining 623 patients. The final response rate in PGA (LOCF) was 80.7% (improved, 60.7%; slightly improved, 20.1%), and the response rates were sustained from 3 months (82.1%) to up to 24 months in more than 80% (Table 4). Similar results were observed among 489 patients who had no history of IFX treatment and at least one ocular attack in the 6 months preceding IFX treatment, with response rates at 3–24 months of 83.1–90.6% (data not shown).

Efficacy in reducing ocular attacks was evaluated in 620 patients of whom 506 had at least one episode of ocular attack in the 6 months preceding IFX therapy and had no history of IFX use. Among these 506 patients, the median number of attacks was 2.0 during the 6 months before initiation of IFX (at 0 months). This improved significantly to 0.0 during the first 6 months post dosing (at 6 months, *p* < 0.001), and the proportion of patients experiencing ocular attacks during the 6-month post-dose period decreased from 100% to 30.5% (Table 3). These figures remained almost constant throughout the study period, with 217 patients (42.9%) experiencing no ocular attacks during the 24-month study period. Similar results were obtained on analysis of all patients who underwent evaluation of the number of ocular attacks during the 6 months before IFX (*n* = 620); in these patients also, the number of ocular attacks decreased significantly.

**Table 3:** Efficacy of infliximab in ocular symptoms of Behçet’s disease

	At 0 M	At 6 M	At 12 M	At 18 M	At 24 M	At 24 M (LOCF)
Physician global assessment (PGA)						
Number of evaluated patients^a^	–	*n* = 582	*n* = 557	*n* = 485	*n* = 465	*n* = 623
Improved	–	359 (61.7)	356 (63.9)	309 (63.7)	294 (63.2)	378 (60.7)
Slightly improved	–	127 (21.8)	108 (19.4)	93 (19.2)	95 (20.4)	125 (20.1)
Unchanged	–	87 (14.9)	84 (15.1)	70 (14.4)	67 (14.4)	105 (16.9)
Worsened	–	9 (1.5)	9 (1.6)	13 (2.7)	9 (1.9)	15 (2.4)
Ocular attacks						
Number of evaluated patients^b^	*n* = 620	*n* = 613	*n* = 578	*n* = 540	*n* = 540	*n* = 620
number of ocular attacks per 6 M	2.0 (1.0, 4.0)	0.0 (0.0, 1.0)	0.0 (0.0, 1.0)	0.0 (0.0, 1.0)	0.0 (0.0, 0.0)	0.0 (0.0, 1.0)^d^
rate of patients with ocular attack per 6 M	542 (87.4)	168 (27.4)	184 (31.8)	144 (26.7)	132 (24.4)	157 (25.3)^d^
Number of evaluated patients^c^	*n* = 506	*n* = 502	*n* = 479	*n* = 448	*n* = 448	*n* = 506
number of ocular attacks per 6 M	2.0 (2.0, 4.0)	0.0 (0.0, 1.0)	0.0 (0.0, 1.0)	0.0 (0.0, 1.0)	0.0 (0.0, 1.0)	0.0 (0.0, 1.0)^d^
rate of patients with ocular attack per 6 M	506 (100.0)	153 (30.5)	169 (35.3)	135 (30.1)	123 (27.5)	146 (28.9)^d^

Data are number (percentage, or median (1st quartile, 3rd quartile). Efficacy data at 0 months (M) to 24 M were analyzed as-observed, and those at 24 M (last observation carried forward (LOCF)) were analyzed using the LOCF method

^a^Evaluated in 623 patients in whom PGA data were available excluding “underminable”

^b^Evaluated in 620 patients in whom number of ocular attacks within 6 months prior to infliximab treatment were evaluated

^c^Evaluated in 506 patients with at least one episode of ocular attacks in the prior 6 months and no history of infliximab treatment

^d^Ocular attacks for 6 months during last observation period

**Fig. 2:**
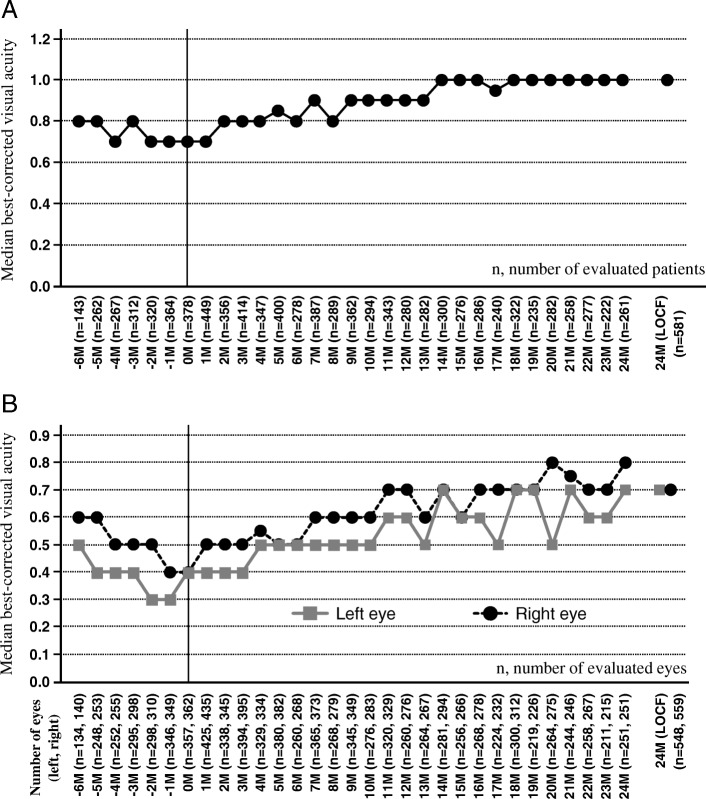
Change in the best-corrected visual acuity per patient (defined as better corrected acuity at each time point per patient) (**a**), and per each eye (**b**). The corrected visual acuity was examined on a monthly basis. *M* months, *LOCF* last observation carried forward

Best-corrected visual acuity in 581 patients before and during IFX therapy is shown in Fig. 2a. Median best-corrected visual acuity before IFX treatment was 0.7–0.8 and was 0.9–1.0 at 9–24 months, showing the maintenance of corrected visual acuity levels. Results were similar for analysis in each eye (left, *n* = 548; right, *n* = 549) (Fig. 2b).

### Reduction in concomitant drugs

Table 4 shows the patient rate and dose of concomitant use of cyclosporine, oral glucocorticoids, and colchicine during this study. Although marked dose reductions in these drugs were not observed, usage rates gradually decreased during the study.

**Table 4:** Percentage and dosage of concomitant drug use

	Day 0 (*n* = 656)	Day 180 (*n* = 624)	Day 365 (*n* = 597)	Day 545 (*n* = 565)	Day 730 (*n* = 544)
Cyclosporine					
*n* (rate)	178 (27.1)	121 (19.4)	112 (18.8)	103 (18.2)	99 (18.2)
dose, mg/day	150.00 (100.00, 200.00)	100.00 (100.00, 150.00)	100.00 (75.00, 150.00)	100.00 (75.00, 150.00)	100.00 (75.00, 150.00)
Oral glucocorticoids					
*n* (rate)	154 (23.5)	109 (17.5)	100 (16.8)	95 (16.8)	89 (16.4)
dose, mg/day	10.00 (7.50, 20.00)	6.50 (5.00, 10.00)	5.00 (4.00, 10.00)	5.00 (5.00, 10.00)	5.00 (5.00, 10.00)
Colchicine					
*n* (rate)	271 (41.3)	202 (32.4)	186 (31.2)	168 (29.7)	155 (28.5)
dose, mg/day	1.00 (1.00, 1.00)	1.00 (0.50, 1.00)	1.00 (0.50, 1.00)	1.00 (0.50, 1.00)	1.00 (0.50, 1.00)

Data are number (percentage) or median (1st quartile, 3rd quartile)

### Associated factors for safety and efficacy

To investigate background factors that may influence the incidence of infections and efficacy, univariate analysis was first performed to check for correlations with various factors. Independent associated factors were then identified using multivariate logistic regression analysis.

Analysis of associated factors in infections was performed in all 656 patients included in the safety assessment. Table 5 shows the results of univariate analysis of patient background factors related to safety and efficacy (PGA response or absence of ocular attacks). Univariate analysis revealed that patients with a history of allergic disease or comorbid respiratory disease had a significantly higher incidence of infections (Table 5). In addition to these two factors, multivariate analysis also identified concomitant glucocorticoids as an independent associated factor.

**Table 5:** Association between patient background and occurrence of infections, PGA response, and reduction in ocular attacks

	Infections			PGA response			Reduction in ocular attacks		
	All	Occurred	*p* ^a^	All^b^	Response rate^c^	*p* ^a^	All^d^	Absence of ocular attacks^e^	*p* ^a^
	*n*	*n* (%)		*n*	*n* (%)		*n*	*n* (%)	
All patients	656	78 (11.89)	–	623	503 (80.7)	–	506	217 (42.9)	–
Disease duration of BD, years									
< 5	268	25 (9.33)	0.130	255	216 (84.7)	0.008	212	88 (41.5)	0.216
≥ 5–< 10	159	17 (10.69)		153	128 (83.7)		129	62 (48.1)	
≥ 10–< 15	95	17 (17.89)		88	70 (79.5)		73	24 (32.9)	
≥ 15	77	11 (14.29)		74	50 (67.6)		55	23 (41.8)	
unknown	57	8 (14.04)		53	39 (73.6)		37	20 (54.1)	
Disease duration of uveitis, years									
< 5	294	28 (9.52)	0.080	274	234 (85.4)	0.030	234	99 (42.3)	0.011
≥ 5–< 10	166	19 (11.45)		162	132 (81.5)		130	70 (53.8)	
≥ 10–< 15	86	17 (19.77)		82	66 (80.5)		71	22 (31.0)	
≥ 15	60	7 (11.67)		58	40 (69.0)		41	15 (36.6)	
unknown	50	7 (14.00)		47	31 (66.0)		30	11 (36.7)	
Severity of ocular symptoms									
severe	408	53 (12.99)	0.342	390	326 (83.6)	< 0.001	332	125 (37.7)	0.007
moderate	201	18 (8.96)		193	157 (81.3)		154	81 (52.6)	
mild	36	4 (11.11)		33	18 (54.5)		14	7 (50.0)	
unknown	11	3 (27.27)		7	2 (28.6)		6	4 (66.7)	
History of allergic disease									
no	618	69 (11.17)	0.034	586	472 (80.5)	0.830	480	206 (42.9)	1.000
yes	38	9 (23.68)		37	31 (83.8)		26	11 (42.3)	
Extraocular symptoms of BD									
oral aphthous ulcers^f^									
no	117	11 (9.40)	0.432	112	81 (72.3)	0.016	82	34 (41.5)	0.808
yes	534	66 (12.36)		506	419 (82.8)		421	182 (43.2)	
skin lesions^f^									
no	259	30 (11.58)	0.902	245	186 (75.9)	0.012	197	76 (38.6)	0.118
yes	392	47 (11.99)		373	314 (84.2)		306	140 (45.8)	
central nervous system lesions^f^									
no	587	68 (11.58)	0.542	559	463 (82.8)	0.001	460	197 (42.8)	0.873
yes	64	9 (14.06)		59	37 (62.7)		43	19 (44.2)	
intestinal tract lesions^f^									
no	584	67 (11.47)	0.423	557	460 (82.6)	0.003	471	200 (42.5)	0.462
yes	67	10 (14.93)		61	40 (65.6)		32	16 (50.0)	
Comorbidity									
respiratory disease									
no	646	74 (11.46)	0.022	614	497 (80.9)	0.385	500	215 (43.0)	0.704
yes	10	4 (40.00)		9	6 (66.7)		6	2 (33.3)	
cardiac disease									
no	646	76 (11.76)	0.338	616	500 (81.2)	0.029	499	213 (42.7)	0.469
yes	10	2 (20.00)		7	3 (42.9)		7	4 (57.1)	
diabetes mellitus									
no	625	74 (11.84)	0.778	593	486 (82.0)	0.002	486	214 (44.0)	0.010
yes	31	4 (12.90)		30	17 (56.7)		20	3 (15.0)	
Concomitant drug									
cyclosporine									
no	443	48 (10.84)	0.247	418	338 (80.9)	0.914	332	158 (47.6)	0.003
yes	213	30 (14.08)		205	165 (80.5)		174	59 (33.9)	
glucocorticoids									
no	124	10 (8.06)	0.167	114	101 (88.6)	0.018	93	43 (46.2)	0.488
yes	532	68 (12.78)		509	402 (79.0)		413	174 (42.1)	

The association between the safety/efficacy and patient background factors (as described in Table 1) such as sex, age, and comorbidity were evaluated using univariate analysis. Results were shown in the patient background with significant (*p* < 0.05) association with occurrence of infections, physician global assessment (PGA) response, or reduction in ocular attacks

^a^Statistical difference was evaluated using chi-square test or Fisher’s exact test, excluding the “unknown” patients

^b^Patients with available PGA data excluding “underminable”

^c^Response was defined as improved or slightly improved

^d^Evaluated in 506 patients who showed at least one ocular attacks in prior 6 months and had no history of infliximab treatment

^e^Patients with absence of ocular attacks during the study period

^f^*n* = 643 (excluded the 5 patients without data on extraocular symptoms of Behçet’s disease (BD))

Meanwhile, age was not significantly associated with infections or serious infections (data not shown), and no difference was observed in the incidence of infections and serious infections between elderly (*≥* 65 years, *n* = 25) (16.00%, 4.00%) and non-elderly subjects (< 65 years, *n* = 631) (11.73%, 3.65%, respectively). Moreover, incidence of infections showed no significant increase in patients receiving concomitant cyclosporine.

Disease duration, severity of ocular symptoms, and comorbid diabetes mellitus were significantly associated with both PGA response and the absence of ocular attacks. The efficacy of IFX was significantly lower in patients with longer disease duration and those with comorbid diabetes mellitus. Meanwhile, in patients with severe ocular symptoms, efficacy was higher than those in patients with moderate/mild symptoms (83.6% versus 77.4%) in terms of PGA response, but lower in terms of absence of ocular attacks (37.7% versus 52.4%). In addition, while some extraocular symptoms (oral aphthous ulcers, skin lesions, central nervous system lesions, and intestinal tract lesions) were significantly associated with PGA responses, no extraocular symptoms were significantly associated with occurrence of ocular attacks. The results of multivariate analysis are shown in Additional file 1: Table S1. Some extraocular symptoms (oral aphthous ulcers, central nervous system lesions, and intestinal tract lesions) and some comorbidities (cardiac diseases and diabetes mellitus) were identified as associated factors for PGA response, whereas ocular symptom severity, number of ocular attacks during the 6 months preceding IFX treatment, and other factors were identified as independent associated factors related to the absence of ocular attacks.

## Discussion

The number of patients with BD in Japan is estimated to be approximately 20,000 (number of persons with specific (intractable) disease healthcare certificates by disease and sex: http://www.mhlw.go.jp/english/database/db-hh/xls/2-22.xls, last accessed December 25, 2018), 60% of whom have pre-existing ocular lesions. Further, 10% of these patients are unresponsive to conventional therapy [20].

Japan is the first country in the world to approve IFX for clinical use against RUBD (https://www.mt-pharma.co.jp/e/release/nr/tanabe/2007/pdf/20070126e.pdf, last accessed December 25, 2018), and the efficacy and safety of this therapy have been previously demonstrated in patients with RUBD in Japan and in other countries [21–30]. However, the sample sizes in these studies were relatively limited, and factors associated with safety and efficacy were not fully clarified. Accordingly, the present study adds to the literatures [21–30] by documenting the safety and efficacy profile of IFX in 656 (650) patients with RUBD, a large population, over an extended time period.

The incidence of any ADR and serious ADRs during the first 6 months (18.60% and 2.90%) were not higher than those in 6-month Japanese PMS studies in patients with rheumatoid arthritis (28.02% and 6.16%) and psoriasis (22.51% and 6.94%, respectively) (Additional file 1: Table S2) [31, 32]. The most common ADR classified by SOC was “Infections and infestations”, which is consistent with findings in the above studies.

Some studies reported that infection was more frequent soon after the start of treatment with TNF inhibitors [33–35], and the same tendency was noted in the present study. However, infections were observed throughout the study period, indicating that attention should be paid to infections not only soon after treatment initiation but also thereafter.

Patients with comorbid respiratory disease or history of allergic disease had a significantly higher incidence of infections. In addition to these two factors, concomitant use of glucocorticoids was also identified as an independent factor associated with infection (Additional file 1: Table S1). Comorbid respiratory disease and concomitant glucocorticoid use were identified as risk factors for infection in Japanese PMS when using IFX and other biologic agents [36–38]. In addition, a history of allergic disease was reported as a risk factor for infections in a UK cohort study [39]. Patients with these risk factors warrant particular attention to risk of infections. Although age and concomitant cyclosporine use were not significantly correlated with infections in this study, advanced age and concomitant use of immunosuppressants are reported risk factors for serious infections in other diseases [36–38]. Patients with such risk factors therefore warrant more careful monitoring.

TB, a major concern during treatment with TNF inhibitors [40, 41], was observed in only two patients (0.30%) during the 24-month follow-up period. However, this occurrence is higher than that in the Japanese population (14.4/100,000 patient-years,) (http://www.mhlw.go.jp/file/06-Seisakujouhou-10900000-Kenkoukyoku/0000133822.pdf, Japanese article, last accessed December 25, 2018), despite 36.9% of patients receiving prophylactic anti-TB drugs. Careful attention therefore should be paid to TB during IFX treatment.

IRs were observed in 11.13% of patients; however, most were non-serious. After the occurrence of their first IR, most patients were re-treated with IFX; serious IRs were observed in approximately 3% of patients at re-infusion, whereas two thirds had no further IR (Additional file 1: Figure S1). Re-infusion therefore appears to be relatively safe in patients with a history of IRs.

In this study, IFX exhibited good efficacy as observed in a previous Japanese clinical trial [18], showing rapid and long-term PGA response, decreasing number of ocular attacks, and maintenance of visual acuity. In addition, the rates of cyclosporine, glucocorticoid, and colchicine use were also reduced after initiation of IFX treatment. ADRs related to cyclosporine such as renal dysfunction and central nervous system effects limit the therapeutic benefit of cyclosporine [42, 43]. IFX treatment enabled a reduction in the use of immunosuppressants, and therefore potentially reduced the risk of ADRs related to these agents.

We also identified patient background factors associated with efficacy (PGA responses and absence of ocular attacks). The incidence of ocular attacks following administration of IFX was lower in patients with severe ocular symptoms than in those with moderate/mild symptoms. Interestingly, however, the PGA response rate was conversely higher. PGA responses represent a subjective measure, and efficacy may have been evaluated higher in those patients with more severe ocular symptoms. In addition, some extraocular symptoms were identified as factors associated with PGA responses. The responsiveness of these symptoms to IFX treatment is also conjectured to have influenced the evaluation of PGA responses.

In this study, the number of ocular attacks during the 6 months just prior to IFX treatment was identified as independently negatively associated with the absence of ocular attacks following IFX treatment (odds ratio = 0.757, Additional file 1: Table S2). The annual number of ocular attacks and cumulative number of ocular attacks in RUBD patients have been reported to be associated with reductions in visual acuity [44, 45]. Visual acuity was maintained in patients with early introduction of IFX therapy [25]. The results of univariate analysis in the present study also showed that patients with a shorter duration of uveoretinitis had a significantly lower incidence of ocular attacks and greater improvement in PGA following administration of IFX. Taking these findings into account, early introduction of IFX therapy prior to severe progression of uveoretinitis is expected to lead to a reduction in ocular attacks and the maintenance of visual acuity.

IFX treatment has also been reported to have excellent efficacy in the treatment of several extraocular symptoms of BD [46–48]. In August 2015 the indication of IFX for intestinal, neurological, and vascular involvement in Behçet’s disease was approved in Japan, based on the results of a phase III study [49]. PMS in these patients is now ongoing in Japan, and the results are expected to provide new insights into BD treatment with IFX.

## Conclusions

The BRIGHT study, a prospective large-scale, long-term PMS study, has clarified the long-term safety and efficacy of and adherence to IFX treatment, and identified associated factors for safety and efficacy, in the treatment of RUBD patients in real-world clinical settings. The safety profile of IFX was similar to that observed in previous studies, and no new safety concerns were observed. In addition, the efficacy of IFX treatment was maintained for an extended period. These results suggest that IFX is a suitable treatment option for RUBD in real-world clinical settings.

## Additional file

Additional file 1: **Figure S1.** Re-infusion of IFX in patients with BD who developed IRs. **Table S1.** Independent factors associated with any infections and efficacy. **Table S2.** Safety profile of IFX therapy in patients with BD, RA, Crohn’s disease (CD) or psoriasis (PsO) in the Japanese PMS study. (PDF 151 kb)

## Abbreviations

ADRs: Adverse drug reactions
BD: Behçet’s disease
CI: Confidence interval
EAU: Experimental autoimmune uveoretinitis
GPSP: Good post-marketing surveillance practice
HBV: Hepatitis B virus
IFX: Infliximab
IR: Infusion reactions
LOCF: Last observation carried forward
MedDRA: Medical dictionary for regulatory activities
PGA: Physician global assessment
PMS: Postmarketing surveillance
Q1: First quartile
Q3: Third quartile
RUBD: Refractory uveoretinitis in Behçet’s disease (RUBD)
SOC: System organs class
TB: Tuberculosis
TNF: Tumor necrosis factor-alpha
TST: Tuberculin skin tests
