# Supplementary material for: Safety and efficacy of infliximab in the treatment of refractory uveoretinitis in Behçet’s disease: a large-scale, long-term postmarketing surveillance in Japan
Source: Arthritis Res Ther. 2019 Jan 5;21:2. doi: 10.1186/s13075-018-1793-7 (PMC6321670; doi:10.1186/s13075-018-1793-7)
Supplement: Supplementary file 1 — Figure S1. Re-infusion of IFX in patients with BD who developed IRs. Table S1. Independent factors associated with any infections and efficacy. Table S2. Safety profile of IFX therapy in patients with BD, RA, Crohn’s disease (CD) or psoriasis (PsO) in the Japanese PMS study. (PDF 151 kb) [file 13075_2018_1793_MOESM1_ESM.pdf]

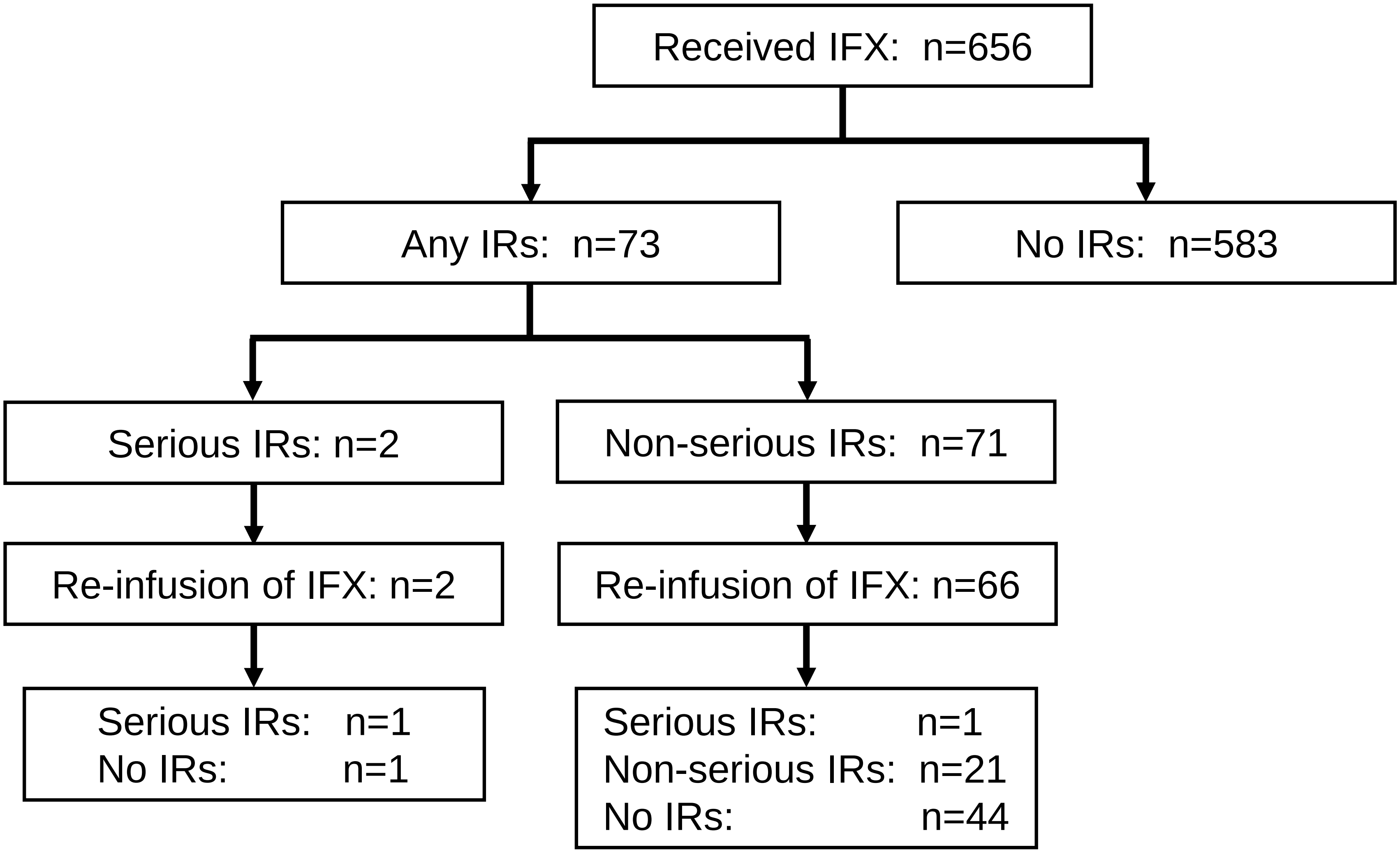

Figure S1. Re-infusion of IFX in BD patients who developed IRs

BD, Behçet’s disease; IFX, infliximab; IRs, infusion reactions.

Table S1. Independent associated factors for any infections and efficacy

Independent associated factors for any infections

	Odds ratio	95% CI	p
History of allergic disease *	2.959	1.254–6.985	0.0133
Comorbid respiratory diseases *	8.275	1.600–42.790	0.0117
Concomitant oral glucocorticoids use *	1.839	1.090–3.104	0.0225

Independent associated factors for PGA response (improved or slightly improved)

	Odds ratio	95% CI	p
Extraocular symptoms of BD			
oral aphthous ulcers *	2.097	1.224–3.590	0.0070
central nervous system lesions *	0.321	0.173–0.595	0.0003
intestinal tract lesions *	0.433	0.221–0.848	0.0147
Comorbidity			
cardiac disease *	0.176	0.036–0.863	0.0323
diabetes mellitus *	0.374	0.150–0.929	0.0342

Independent associated factors for absence of ocular attacks after IFX treatment

	Odds ratio	95% CI	p
Severity of ocular symptoms (severe versus moderate/mild)	0.596	0.392–0.907	0.0157
Number of ocular attacks in prior 6 months (per 1 attack)	0.757	0.672–0.853	<0.0001
Extraocular symptoms of BD in skin lesions *	1.537	1.015–2.330	0.0425
Comorbid kidney disease *	3.802	1.235–11.707	0.0200
Concomitant cyclosporine use *	0.671	0.450–1.000	0.0498

* Yes versus no. Multiple logistic regression analysis was performed to identify independent associated factors for development of any infections, response in PGA (improved or slightly improved); the explanatory variables used were sex, age, disease duration of BD, disease duration of uveoretinitis, severity of ocular symptoms, history of allergic disease, history of IFX treatment, extraocular symptoms of BD, comorbidities, concomitant drugs use (cyclosporine, glucocorticoids, and colchicine). In multiple logistic regression analysis for absence of ocular attacks after initiation of IFX treatment, number of ocular attacks in 6 months prior to IFX treatment was included as explanatory variables in addition to above variables. In all multiple logistic regression analysis, a stepwise selection process was used. PGA, physician global assessment; BD, Behçet's disease; IFX, infliximab.

Table S2. Safety profile of IFX therapy in patients with BD, RA, CD or PsO in Japanese PMS study

	BD-PMS (Day 0-180) (n=656)	BD-PMS (Day 0-730) (n=656)	RA-PMS * (6 months) (n=5,000)	CD-PMS † (1 year) (n=653)	PsO-PMS ‡ (6 months) (n=764)
Any ADRs	122 (18.60)	212 (32.32)	1401 (28.02)	93 (14.24)	172 (22.51)
Blood and lymphatic system disorders	0	1 (0.15)	9 (0.18)	2 (0.31)	3 (0.39)
Cardiac disorders	2 (0.30)	3 (0.46)	28 (0.56)	1 (0.15)	1 (0.13)
Ear and labyrinth disorders	0	0	2 (0.04)	0	1 (0.13)
Endocrine disorders	0	0	1 (0.02)	0	0
Eye disorders	3 (0.46)	6 (0.91)	7 (0.14)	0	1 (0.13)
Gastrointestinal disorders	8 (1.22)	12 (1.83)	124 (2.48)	16 (2.45)	13 (0.70)
General disorders and administration site conditions	15 (2.29)	31 (4.73)	275 (5.50)	7 (1.07)	29 (3.80)
Hepatobiliary disorders	4 (0.61)	5 (0.76)	91 (1.82)	1 (0.15)	13 (1.70)
Immune system disorders	2 (0.30)	3 (0.46)	10 (0.20)	1 (0.15)	2 (0.26)
Infections and infestations	35 (5.34)	78 (11.89)	433 (8.66)	20 (3.06)	39 (5.10)
Injury, poisoning and procedural complications	7 (1.07)	19 (2.90)	–	17 (2.60)	30 (3.93)
Investigations	18 (2.74)	30 (4.57)	223 (4.46)	9 (1.38)	27 (3.53)
Metabolism and nutrition disorders	1 (0.15)	2 (0.30)	8 (0.16)	1 (0.15)	1 (0.13)
Musculoskeletal, and connective tissue disorders	3 (0.46)	8 (1.22)	38 (0.76)	8 (1.23)	17 (2.23)
Neoplasm benign, malignant, and unspecified	1 (0.15)	3 (0.46)	9 (0.18)	1 (0.15)	6 (0.79)
Nervous system disorders	7 (1.07)	9 (1.37)	191 (3.82)	6 (0.92)	14 (1.83)
Psychiatric disorders	0	0	3 (0.06)	0	1 (0.13)

Renal and urinary disorders	0	0	12 (0.24)	0	0
Reproductive system and breast disorders	0	0	2 (0.04)	0	1 (0.13)
Respiratory, thoracic, and mediastinal disorders	12 (1.83)	29 (4.42)	141 (2.82)	8 (1.23)	11 (1.44)
Skin & subcutaneous tissue disorders	39 (5.95)	63 (9.60)	317 (6.34)	25 (3.83)	39 (5.10)
Vascular disorders	0	0	115 (2.30)	2 (0.31)	2 (0.26)
Serious ADRs	19 (2.90)	40 (6.10)	308 (6.16)	27 (4.13)	53 (6.94)
Blood and lymphatic system disorders	0	0	6 (0.12)	0	2 (0.26)
Cardiac disorders	1 (0.15)	1 (0.15)	5 (0.10)	1 (0.15)	0
Ear and labyrinth disorders	0	0	0	0	0
Endocrine disorders	0	0	1 (0.02)	0	0
Eye disorders	1 (0.15)	2 (0.30)	0	0	0
Gastrointestinal disorders	1 (0.15)	1 (0.15)	13 (0.26)	7 (1.07)	3 (0.39)
General disorders and administration site conditions	0	2 (0.30)	26 (0.52)	2 (0.31)	8 (1.05)
Hepatobiliary disorders	0	0	1 (0.02)	0	3 (0.39)
Immune system disorders	0	1 (0.15)	10 (0.20)	1 (0.15)	1 (0.13)
Infections and infestations	11 (1.68)	24 (3.66)	202 (4.04)	9 (1.38)	19 (2.49)
Injury, poisoning and procedural complications	1 (0.15)	1 (0.15)	–	3 (0.46)	7 (0.92)
Investigations	0	1 (0.15)	14 (0.28)	2 (0.31)	6 (0.79)
Metabolism and nutrition disorders	0	0	0	0	0
Musculoskeletal, and connective tissue disorders	0	1 (0.15)	6 (0.12)	5 (0.77)	3 (0.39)
Neoplasm benign, malignant, and unspecified	1 (0.15)	2 (0.30)	9 (0.18)	1 (0.15)	4 (0.52)
Nervous system disorders	1 (0.15)	2 (0.30)	8 (0.16)	0	2 (0.26)
Psychiatric disorders	0	0	1 (0.02)	0	0

Renal and urinary disorders	0	0	1 (0.02)	0	0
Reproductive system and breast disorders	0	0	1 (0.02)	0	0
Respiratory, thoracic, and mediastinal disorders	3 (0.46)	4 (0.61)	40 (0.80)	2 (0.31)	5 (0.65)
Skin & subcutaneous tissue disorders	1 (0.15)	1 (0.15)	8 (0.16)	2 (0.31)	4 (0.52)
Vascular disorders	0	0	3 (0.06)	1 (0.15)	0

Data are number (%). * RA-PMS, PMS of IFX in Japanese patients with RA (MedDRA/J version 7.1); † CD-PMS, PMS of IFX in Japanese patients with CD (MedDRA/J, version 14.1); ‡ PsO-PMS; PMS of IFX in Japanese patients with PsO (MedDRA/J version 16.1). IFX, infliximab; ADRs, adverse drug reactions; BD, Behçet’s disease; RA, rheumatoid arthritis; CD, Crohn's disease; PsO, psoriasis; PMS, postmarketing surveillance.

RA-PMS: Takeuchi T, et al. Ann Rheum Dis 2008; 67: 189–94.

PsO-PMS: Torii H, et al. J Dermatol 2016; 43: 767–78.

CD-PMS: Mitsubishi Tanabe Pharma Corporation internal data.
